# Structural Characterization of a Recombinant Fusion Protein by Instrumental Analysis and Molecular Modeling

**DOI:** 10.1371/journal.pone.0057642

**Published:** 2013-03-04

**Authors:** Zhigang Wu, Peng Zhou, Xiaoxin Li, Hui Wang, Delun Luo, Huaiyao Qiao, Xiao Ke, Jian Huang

**Affiliations:** 1 Chengdu Kanghong Biotechnology Inc., Chengdu, P. R. China; 2 Center of Bioinformatics, University of Electronic Science and Technology of China, Chengdu, P. R. China; 3 Peking University People’s Hospital, Beijing, P. R. China; 4 State Key Laboratory of Pathogens and Biosecurity, Beijing Institute of Microbiology and Epidemiology, Beijing, P. R. China; Dundee University, United Kingdom

## Abstract

Conbercept is a genetically engineered homodimeric protein for the treatment of wet age-related macular degeneration (wet AMD) that functions by blocking VEGF-family proteins. Its huge, highly variable architecture makes characterization and development of a functional assay difficult. In this study, the primary structure, number of disulfide linkages and glycosylation state of conbercept were characterized by high-performance liquid chromatography, mass spectrometry, and capillary electrophoresis. Molecular modeling was then applied to obtain the spatial structural model of the conbercept–VEGF-A complex, and to study its inter-atomic interactions and dynamic behavior. This work was incorporated into a platform useful for studying the structure of conbercept and its ligand binding functions.

## Introduction

Age-related macular degeneration (AMD) is the leading cause of irreversible blindness among people who are 50 years of age or older in the developed world and is one of the three major causes of blindness in developing countries [1], [2]. In exudative (wet) AMD, the choroidal neovascularization (CNV) is believed to be the cause of the severely progressive decrease of central visual acuity [3]. Vascular endothelial growth factor (VEGF), a major factor of angiogenesis and vascular permeability implicated in the development of the CNV, is highly expressed in AMD of different forms and stages. VEGF is also an important therapeutic target for treatment of CNV resulting from AMD [4], [5], [6], [7], [8], [9].

Several VEGF antagonists have been developed for treatment of wet AMD including Pegaptanib (Macugen; Eyetech Pharmaceuticals, Inc., New York, NY, USA), Ranibizumab (Lucentis; Genentech, Inc., San Francisco, CA, USA), and VEGF Trap-Eye (Regeneron Pharmaceuticals, Inc., New York, NY, USA). Conbercept is a recombinant fusion protein designed as a receptor decoy with high affinity for all VEGF isoforms and placental growth factors (PlGF). It is composed of human VEGF receptor 1 (VR1 or Flt-1) domain 2 and human VEGF receptor 2 (VR2 or KDR) domains 3, 4 and the Fc portion of human IgG1, which serves to create a homodimer of the fusion protein. The multi-component design of conbercept makes structural characterization and development of a functional assay challenging. In this study, we report the extensive structural characterization of conbercept using different analytical techniques. Molecular modeling was applied for exploring the interaction profile of the conbercept–VEGF-A complex, and for analyzing the effect of structural variations on its binding and interactions. This should provide deeper insight into the molecular mechanism and structural factors determining conbercept binding behavior to VEGF-A and other target proteins.

## Materials and Methods

### 1. Reagents

The conbercept protein used in this study was produced from Chinese hamster cells at Chengdu Kanghong Biotechnology Co. Ltd. ProteoExtract™ All-in-One Trypsin Digestion Kit (including extraction buffer, digest buffer, reducing agent, blocking agent, trypsin) was purchased from Merck KGaA (Darmstadt, Germany). PNGase F was purchased from New England BioLabs (Hitchin, Hertfordshire, UK). The Signal™ 2-AA Labeling Kit was purchased from Prozyme (Hayward, CA, USA). The MS-grade acetonitrile was purchased from Sigma–Aldrich (St. Louis, MO, USA). The water was purified using a Millipore Milli-Q Gradient Water Purification System (Billerica, MA, USA). All other chemicals, unless otherwise stated, were purchased from Sigma.

### 2. Capillary Electrophoresis System

A PA800 plus (Beckman Coulter, Brea, CA, USA) capillary electrophoresis system was used for protein isoelectric point (pI) analysis. Sample buffer was prepared by mixing pI standard markers with a desalted protein sample. The sample running buffer solutions were prepared according to the manufacturer’s protocol. The sample was analyzed and the protein pI was assigned by Karat software.

### 3. High-performance Liquid Chromatography (HPLC) Equipment

Unless otherwise specified, the HPLC system used in this study consisted of an Agilent 1260 (Agilent, Santa Clara, CA, USA) separation module equipped with a column heating compartment, dual UV and fluorescence detector.

### 4. Mass Spectrometry

A LTQ (Thermo Fisher, San Jose, CA, USA) ion trap mass spectrometer was used for peptide mapping, disulfide mapping, glycopeptide characterization and oligosaccharide structure elucidation. The nitrogen gas flow rate and the desolvation temperature were set according to the flow rate. Other instrumental parameters were optimized for maximum sensitivity for each experiment. Oligosaccharide structure analysis was carried out using negative mode electrospray ionization (ESI). All other experiments were carried out in positive mode ESI. The ESI source voltage of the LTQ was set at 5.0 kV, and the capillary temperature was set at 275°C. The mass spectrometer was operated in data dependent mode with dynamic exclusion enabled. In this mode, full scan mass spectra (m/z 500–2000) were acquired first. The MS/MS scan was acquired on the three most abundant peaks in each full scan when the signal exceeded a predefined threshold. In the MS/MS scan, the precursor ions were fragmented by collision-induced dissociation (CID) with 35% relative collision energy.

### 5. Peptide Mapping

A solution of 200 µl of conbercept (2 mg) was buffer-exchanged into 50 mM NH_4_HCO_3_ to a concentration of about 10 mg/mL. A total of 30 µl of extraction buffer was added into a 10 µl aliquot of sample. The mixture was vortexed for 1 min and then centrifuged at 10,000×g for 15 min. A total of 25 µl of the supernatant with 25 µl of digest buffer and 1 µl reducing agent were added to a new labeled tube, and the mixture was incubated for 10 min at 37°C. Afterwards, 1 µl of blocking agent was added for 10 min at room temperature. Next, 1 µl of trypsin was added into the resulting mixture and incubated for 1 h at 37°C. After centrifugation at 10,000×g for 3 min, half of the mixture was transferred into another polypropylene tube with 5 µl PNGase F for 1.5 h at 37°C. A total of 5 µl of the peptide mixtures with and without PNGase F treatment was injected separately into the HPLC–mass spectrometer system for analysis. The peptide mixtures were separated with a C18 column (Agilent Eclipse Plus C18 RRHD 1.8 µm 2.1×150 mm). The column was maintained at 60°C; UV absorbance was monitored at 214 nm and analyzed using ESI LTQ mass spectrometry. Peptides were separated by elution with a gradient of acetonitrile containing 0.1% TFA (phase B) in water with 0.05% TFA (phase A). Gradient conditions were as follows: 0–5 min, 0% B; 5–70 min, 0–40% B; 70–71 min, 40–90% B; 71–105 min, 90% B; 105–110 min, 90-0% B; 110–120 min, 0% B.

### 6. Disulfide Mapping

A solution of 200 µl of conbercept (2 mg) was incubated at 70°C for 30 min in the presence of 6M guanidium hydrochloride. Then the solution was buffer-exchanged with 50 mM ammonium bicarbonate (pH∼8.2) using a Microcon spin column (10 kDa MWCO; Millipore, Bedford, MA, USA). Then trypsin was added to the buffer-exchanged solution at a 1∶20 enzyme to protein weight ratio and incubated at 37°C for 16 h. Digestion was stopped by adding 1% formic acid to pH 2. The resulting peptide mixtures including disulfide-linked di-peptides were injected into the HPLC–mass spectrometer system for analysis. Gradient conditions were the same as for the peptide mapping.

### 7. Oligosaccharides Analysis

After buffer exchange, 200 µl of conbercept (2 mg) in 50 mM ammonium bicarbonate was incubated with PNGase F at 37°C for 24 h. An enzyme to protein ratio of 150 mU enzyme per mg protein was used. Released carbohydrates were labeled with 2-amino-benzoic acid (2-AA) as described by the manufacturer. The purified carbohydrates were reconstituted with 50 µL of HPLC-grade water prior to chromatographic analysis. Oligosaccharide mixtures were injected onto a BEH glycan column (1.7 um 2.1×150 mm, Waters) for separation and quantitative analysis using a fluorescence detector. Oligosaccharides were separated with a gradient of acetonitrile (phase B) in water with 100 mM ammonium formate. The gradient conditions were as follows: 0–38.5 min, 78% B; 38.5–38.6 min, 56% B; 38.6–40.5 min, 56–0% B; 40.5–46.5 min, 0% B; 46.5–52 min, 0–78% B; 52–55 min, 78% B.

### 8. Molecular Modeling

#### Construction of the conbercept/VEGF-A binding region

**Figure 1:**
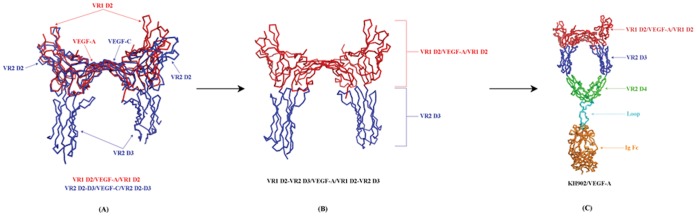
A brief description of the construction of the conbercept–VEGF-A complex model: (A) Alignment of VR1 D2/VEGF-A/VR1 D2 and VR2 D2–D3/VEGF-C/VR2 D2–D3 crystal structures. (**B**) Grafting VR2 D3 from VR2 D2–D3/VEGF-C/VR2 D2–D3 to VR1 D2/VEGF-A/VR1 D2 on the basis of the alignment. (**C**) Adding VR2 D4, loop, and IgG Fc to the VR1 D2-VR2 D3/VEGF-A/VR1 D2-VR2 D3 to obtain the conbercept–VEGF-A complex model.

The crystal structure of VEGF-A in complex with two copies of VR1 D2 (VR1 D2/VEGF-A/VR1 D2) (PDB: 1 flt) was adopted as the starting template to construct the conbercept–VEGF-A complex structure model. This template was first aligned on the complex crystal structure of VEGF-C with two VR2 D2–D3 (VR2 D2–D3/VEGF-C/VR2 D2–D3) (PDB: 2×1 w) to generate a consensus binding mode between the two crystal systems (Figure 1A), and then the VR2 D3 fractions were manually grafted from the VR2 D2–D3/VEGF-C/VR2 D2–D3 to VR1 D2/VEGF-A/VR1 D2 on the basis of the alignment (Figure 1B).

#### Homology modeling of the VR2 D4 structure

To date, an atomically resolved structure of VR2 D4 is still unavailable, thus homology modeling was employed to estimate the VR2 D4 structure. The crystal structure of the titin Ig tandem domain (TITD) (PDB: 3l cy) was used as the 3D coordinate template to perform the modeling. The sequence identity between the template and VR2 D4 is about 33.5%, and thus the homology modeling was feasible. The sequence alignment of VR2 D4 and TITD was carried out using the ClustalW program [10] and *manually adjusted* to ensure *alignment* of important functional residues. The homology model of the VR2 D4 structure was constructed and refined using the MODELLER program as previously described [11]. The resultant structure was evaluated using PROCHECK from the Structure Analysis and Verification Server [12].

#### Protein docking

According to deletion mutant experiments [13] and electron microscopy analyses [14], the receptor dimerization is induced by ligand binding when interacting with VEGF, resulting in nonspecific contact at the D4 region of VR. As a homodimer fusion protein, conbercept contains two identical parts of VR2 D4. Therefore, it is highly possible that self-dimerization might be induced at the D4 region of conbercept when interacting with VEGF-A. A Fourier transform-based method implemented in the Hex program [15] was employed to perform protein docking for the two VR2 D4 parts. The docking output is a list of potential binding modes between the two VR2 D4 parts. These complex candidates were visually examined and one of them was selected to satisfy the geometric constraint and spatial arrangement of the constructed conbercept–VEGF-A binding region. This selected VR2 D4 dimer was then manually added into the conbercept–VEGF-A model.

#### Loop modeling

Subsequently, the IgG Fc and loops of the conbercept–VEGF-A complex were modeled. The IgG Fc was taken from the human IgG1 antibody Fc fragment (PDB: 3 dnk) and the loop regions between the IgG Fc and VR2 D4 were constructed using the SuperLooper server [16], which uses a series of spatial restraints and empirical knowledge to optimize an unstructured polypeptide chain set between two fixed points.

The addition of VR2 D4, loop, and IgG Fc to VR1 D2-VR2 D3/VEGF-A/VR1 D2-VR2 D3 to obtain the conbercept–VEGF-A complex model is shown in Figure 1C.

#### Dynamics simulations

The initial structure of conbercept in complex with VEGF-A was further optimized and refined using a molecular dynamics (MD) procedure to eliminate unreasonable states involved in the model, such as inter-atomic collisions and structural distortions. The MD simulations were performed using the AMBER03 force field [17]. The complex was solvated in a rectangular box with bulk TIP3P water molecules so that the boundary of the box was at least 8 Å away from any solute atom. Particle Mesh Ewald (PME) was employed to study the long-range electrostatic interactions [18]. Following 1,200 steps of minimization, a 50 ps temperature increase from 10 K to 300 K and a 5 ns equilibration, the MD simulation was conducted. The SHAKE procedure was employed to constrain hydrogen atoms [19] during the MD. The equilibrated structure was minimized *via* 2,000 steps; the first 300 steps were performed with the steepest descent algorithm, whereas the rest of the steps were performed with the conjugate gradient algorithm.

## Results

### 1. The Glycosylation Effects on pI

**Figure 2:**
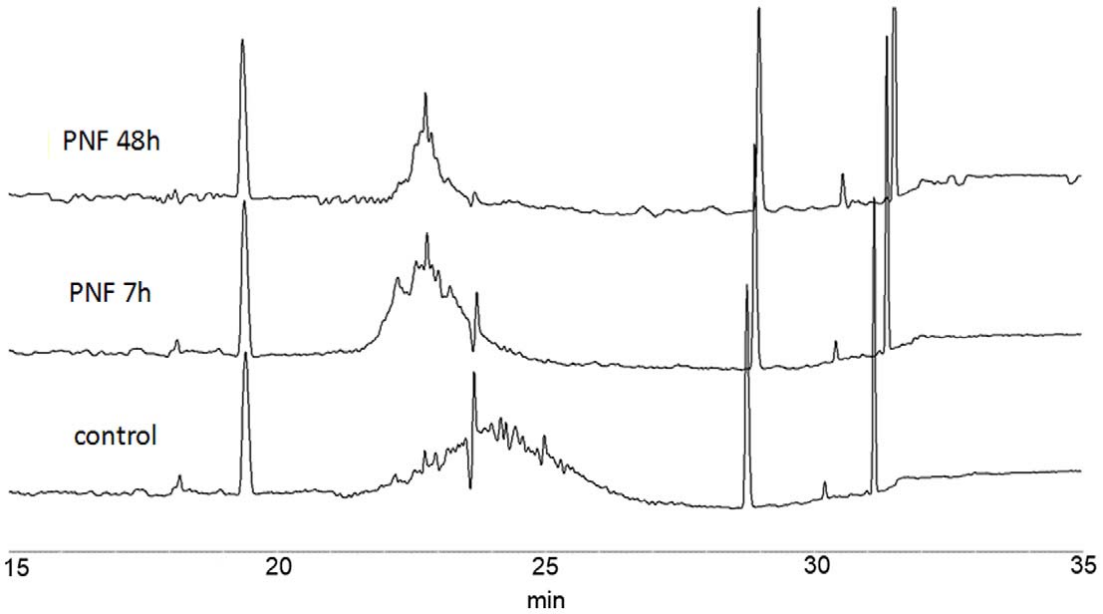
Isoelectric point measurements of conbercept with PNGase F treatment at 0, 7 and 48 h.

The isoelectric point (pI) is the pH at which a molecule or surface carries no net electrical charge. Glycosylation can affect a protein’s pI and lead to heterogeneity because the protein has been differentially glycosylated. The amino acid sequence of conbercept possesses seven potential N-linked glycosylated sites, which makes characterization more complicated. Capillary electrophoresis (CE) is widely used to measure a protein’s pI for its high resolution and linearity over a wide range of pHs [20], [21], [22], [23]. To examine the glycosylation effects on conbercept’s pI, samples of protein treated with PNGase F for 0, 7 and 48 h were analyzed with capillary electrophoresis. As shown in Figure 2, multiple peaks were observed in the untreated sample with a pI range of 5.68–7.47. “Acidic” shifts were observed for the samples treated with PNGase F for 7 and 48 h. The sample became more and more homogeneous with the subsequent removal of glycans.

### 2. Peptide Mapping Results

To confirm its amino acid sequence and obtain detailed structural information such as locations and degree of post-translational modifications, a protein needs to be cleaved into smaller peptides before characterization with mass spectrometer analysis. This is called “peptide fingerprinting” or “peptide mapping” [24], [25], [26], [27], [28], [29], [30]. Peptide mapping allows rapid overall evaluation of a protein’s primary structure from analysis of the peptides produced by enzymatic digestion. Then, the protein can be constructed on the basis of the information obtained from all the peptides identified, and detailed structural information can be obtained through this approach.

**Figure 3:**
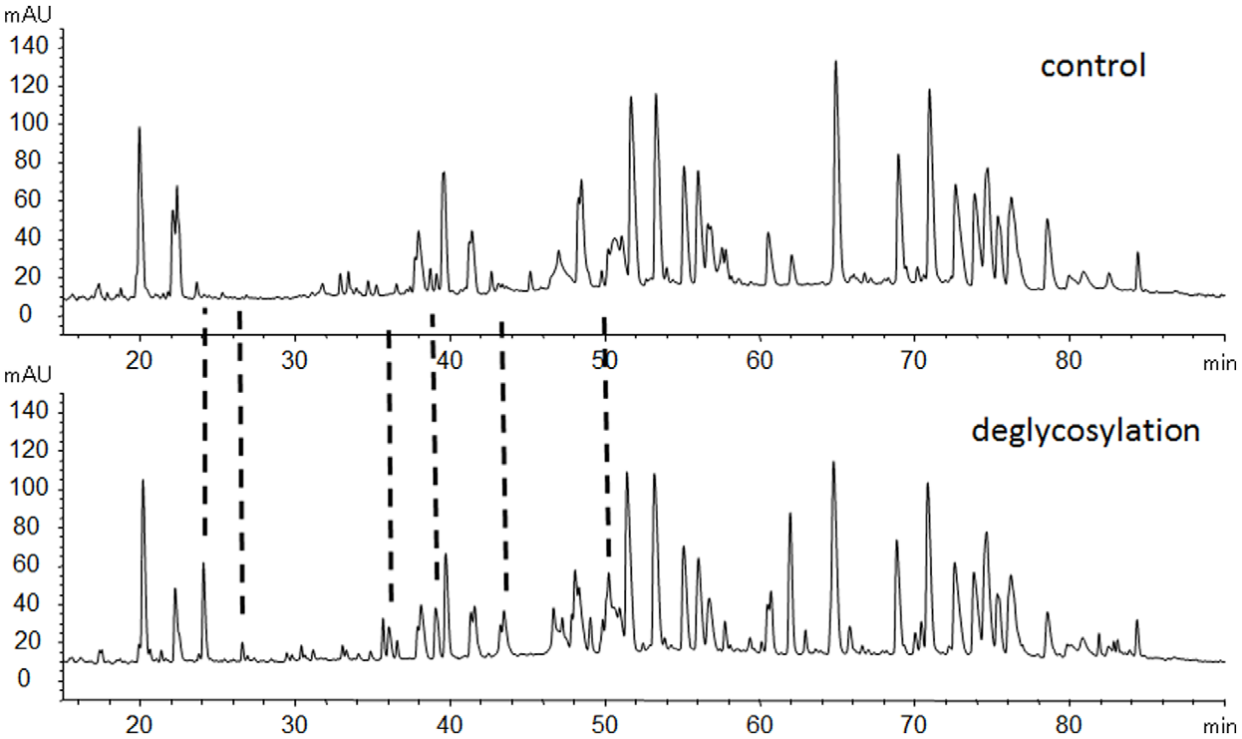
Peptide mapping of conbercept without (up) and with PNGase F treatments (bottom).

Samples of tryptic digested peptide mixtures treated with and without PNGase F were analyzed separately. The annotated UV chromatograms of the LC-MS/MS analysis are presented in Figure 3. The HPLC peaks were assigned peptide sequences by matching to the measured mass of the peptide derived from a simulated trypsin digestion and confirmed by CID MS/MS sequencing. For the sample treated with PNGase F, peptides were identified covering 100% of the sequence. Only very small amounts of peptides with deamidation and oxidation were detected (less than 1% compared with the native forms). These minor modifications were probably introduced during the sample preparation process [31], [32], [33], [34], [35]. Seven peptides containing an N-glycosylation site were found only in the sample treated with PNGase F (as shown in Table 1). This indicates that all seven potential N-glycosylation sites are completely occupied with glycans.

**Table 1:** All seven N-glycosylated tryptic peptides identified with treatment of PNGase F.

Amino acid position	Peptide sequence	MH+ [Da]	Retentiontime (min)
29–39	VTSPNITVTLK	1173.58	50.2
60–69	GFIISNATYK	1114.83	49.8
117–124	LVLNCTAR	947.58	36.0
193–198	NSTFVR	724.50	26.6
242–253	NGIPLESNHTIK	1324.83	39.0
267–281	DTGNYTVILTNPISK	1637.99	61.9
372–380	EEQYNSTYR	1190.58	24.0

### 3. Oligosaccharide Characterization

Two techniques were applied for oligosaccharide characterization: anion-exchange chromatography with fluorescence detection and glycopeptide analysis with HPLC mass spectrometry. In the fluorescence detection approach, oligosaccharides were completely released from the protein by enzymatic digestion, followed by 2-amino-benzoic acid (2-AA) fluorescent derivation, which allows accurate quantification of sub-picomolar levels of glycans [36], [37], [38], [39]. In this experiment, oligosaccharide peaks were isolated and submitted to online analysis by ESI mass spectrometry. Over 20 major oligosaccharide peaks (relative abundance >1%) were identified by matching experimental masses with calculated masses from glycan-2AA labeled structures. They were mainly complex biantennary structures with a core fucose and often terminated with sialic acid residues. To determine the oligosaccharide sequence around each of the seven glycosylation sites, glycopeptide analysis using HPLC mass spectrometry was performed. The protein was digested with trypsin and the peptide mixtures were separated by HPLC and analyzed using mass spectrometry. Glycopeptides usually eluted as a group of relatively poorly resolved peaks because of their lower ionization efficiency. Seven groups of glycopeptide peaks were found that belonged to seven N-glycosylation sites. These peaks were selected and extensively characterized. Each of the seven glycosylation sites was occupied with different oligosaccharide structures, and the three most abundant structures at each site are listed in Table 2.

**Table 2:** All seven *N*-glycosylated tryptic peptides and the major oligosaccharide compositions at each site.

No.	Peptide	Oligosaccharide composition
1	VTSP**N**ITVTLK	(Hex)2(HexNAc)2(Deoxyhexose)1(NeuAc)2+(Man)3(GlcNAc)2
		(Hex)2(HexNAc)2(Deoxyhexose)1(NeuAc)1+(Man)3(GlcNAc)2
		(Hex)2(HexNAc)2(Deoxyhexose)1+(Man)3(GlcNAc)2
2	GFIIS**N**ATYK	(Hex)2(HexNAc)2(Deoxyhexose)1(NeuAc)2+(Man)3(GlcNAc)2
		(Hex)2(HexNAc)2(Deoxyhexose)1(NeuAc)1+(Man)3(GlcNAc)2
		(Hex)2(HexNAc)2(Deoxyhexose)1+(Man)3(GlcNAc)2
3	LVL**N**CTAR	(Hex)2(HexNAc)2(NeuAc)1+(Man)3(GlcNAc)2
		(Hex)2+(Man)3(GlcNAc)2
		(Hex)2(HexNAc)2(NeuAc)2+(Man)3(GlcNAc)2
4	**N**STFVR	(Hex)2(HexNAc)2(NeuAc)1+(Man)3(GlcNAc)2
		(Hex)2(HexNAc)2(Deoxyhexose)1(NeuAc)1+(Man)3(GlcNAc)2
		(Hex)2(HexNAc)2+(Man)3(GlcNAc)2
5	NGIPLES**N**HTIK	(Hex)2(HexNAc)2(Deoxyhexose)1(NeuAc)2+(Man)3(GlcNAc)2
		(Hex)2(HexNAc)2(Deoxyhexose)1(NeuAc)1+(Man)3(GlcNAc)2
		(Hex)2(HexNAc)2(Deoxyhexose)1+(Man)3(GlcNAc)2
6	DTG**N**YTVILTNPISK	(Hex)2(HexNAc)2(Deoxyhexose)1(NeuAc)2+ Man)3(GlcNAc)2
		(Hex)2(HexNAc)2(Deoxyhexose)1(NeuAc)1+(Man)3(GlcNAc)2
		(Hex)2(HexNAc)2(Deoxyhexose)1+(Man)3(GlcNAc)2
7	EEQY**N**STYR	(Hex)1(HexNAc)2(Deoxyhexose)1+(Man)3(GlcNAc)2
		(HexNAc)2(Deoxyhexose)1+(Man)3(GlcNAc)2
		(HexNAc)2+(Man)3(GlcNAc)2

### 4. Disulfide Mapping Results

**Figure 4:**
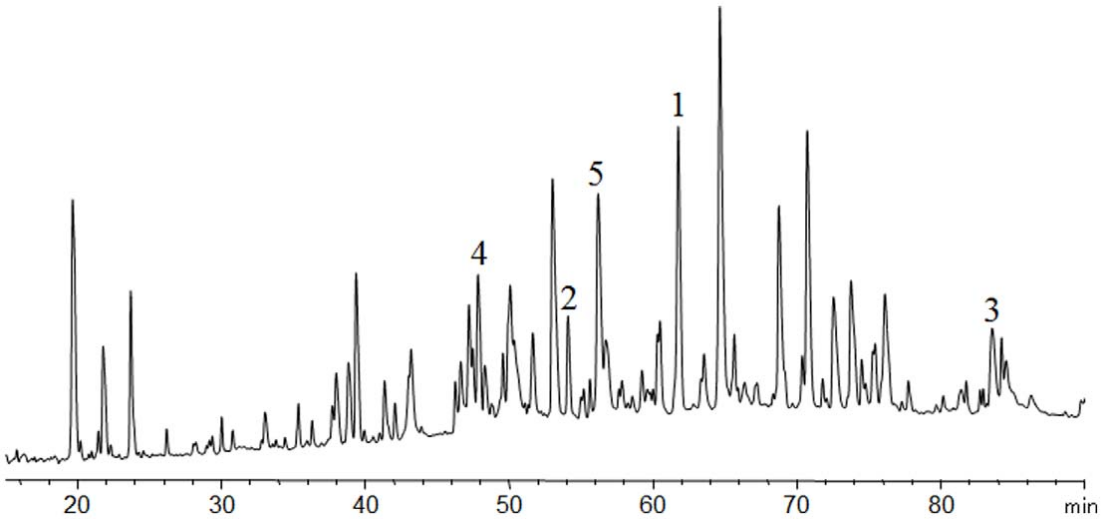
Five peptides with disulfide linkages identified by disulfide mapping.

To determine the disulfide linkages within a protein, the protein must be digested while leaving the disulfide bonds intact [29], [40], [41], [42], [43], [44], [45]. In this experiment, the protein was digested with trypsin without reduction and peptide mixtures were separated by HPLC and analyzed using mass spectrometry. Mass spectrometric analysis was performed to identify the presence of the correct disulfide bonds as well as the absence of incorrect ones. All five disulfide-linked di-peptides were found by matching experimental masses with calculated masses (Figure 4). Based on the MS/MS data of the respective reduced peptide masses, the amino acid sequences of these peptides were determined. Peak 1 corresponds to the di-peptide derived from VR1 domain 2. Peak 2 corresponds to the one derived from VR2 domain 3. Peak 3 corresponds to the homo-di-peptide linked by two disulfide bonds, which is derived from the hinge region of the Fc. Peak 4 corresponds to the one derived from CDR2 region of the Fc. Peak 5 corresponds to the one derived from the CDR3 region of the Fc. In addition, the theoretical masses of all possible combinations for the consequential hypothetical cysteine-linked di-peptides were checked. No mis-pairing of disulfide bonds was detected.

### 5. Structure Model of Conbercept Complexed with VEGF-A

The crude structural model of the conbercept–VEGF-A complex was obtained through molecular modeling procedures as described previously. As shown in Figure 1, this complex can be partitioned into two sections: a functional region composed of VR1 D2, VR2 D3, VR2 D4 and VEGF-A, and an IgG Fc region suspended below the functional region through two flexible loops. The VEGF-A directly touches the juncture of VR1 D2 and VR2 D3 spanning between the two arms of conbercept. In this model, the conbercept–VEGF-A complex exhibits a bridge-like configuration.

## Discussion

Changes in DNA caused by mutation could result in errors in the corresponding protein sequence, which may have diverse effects on protein structure and functions leading to a partially or completely non-functional protein [46]. Fc fusion proteins are normally a target for chemical modifications like oxidation and deamidation, which could cause protein aggregation or degradation. In some cases, such modifications could lead to a loss of protein binding affinity or even bioactivity [47], [48], [49]. For the peptide mapping procedure, the protein was digested before HPLC separation to facilitate peptide identification by mass spectrometry. Successful identification of the digested peptides led to complete sequence recovery and absence of chemical modifications in the antigen binding region. The amino acid sequence confirmation provided convincing evidence that conbercept evolved by successive duplication of precursor genes. Absence of chemical modifications indicated that there were no changes in the chemical nature of the amino acids.

Disulfide linkages play an important role in folding, stability and functions of proteins. Correct disulfide linkages in a therapeutic protein must be confirmed to ensure that the protein is properly produced and folded. Incorrect disulfide linkages lead to protein scrambling and increase product heterogeneity, and may have clinical implications for drug efficacy and safety. Knowledge of disulfide linkages for conbercept is influential in protein folding experiments and in 3-D structure prediction. In this study, two pairs of intermolecular disulfide bonds at the Fc hinge region, one pair of intramolecular disulfide bonds at VR1 domain 2 and VR2 domain 3, respectively, plus two pairs of intramolecular disulfide bonds at the Fc region were identified. The inter-chain disulfide linkages ensured that the protein was a homodimer of two homologous polypeptide chains connected at the Fc hinge region. The intramolecular disulfide bonds ensured that the VR1 domain 2 and VR2 domain 3 kept their original structural features. Mismatched disulfide linkages were excluded by checking all possible combinations for the consequential hypothetical cysteine-linked di-peptides. The availability of the complete disulfide linkages should make structure predictions by molecular modeling more reliable.

**Figure 5:**
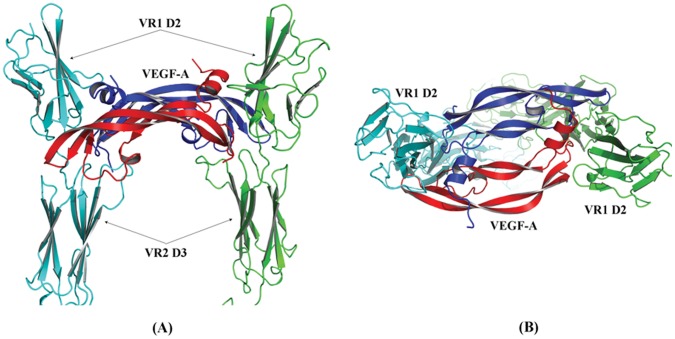
The MD-equilibrated structural model of the conbercept–VEGF-A functional region: (A) side-view, (B) top-view.

Based on the structural information from experimental analysis, the structural and energetic properties of conbercept and its complex with VEGF-A were further examined by molecular modeling. The MD-equilibrated structure model of the conbercept–VEGF-A functional region is shown in Figure 5, in which VEGF-A spans across two arms of conbercept to render a ‘bridge’-like configuration. As anticipated, the complex structure architecture is quite similar to that of VEGF-A bound with its cognate receptor VR1. Nevertheless, according to the SPR assay, the binding affinity of VEGF-A to conbercept is considerably lower than that of its binding to VR1 (0.429 [unpublished data] *vs*∼0.01 nM [50]. This is expected considering that the conbercept heterozygote may not match VEGF-A perfectly as compared with the cognate receptor VR1, since conbercept was designed for trapping not only VEGF-A, but also other isoforms such as VEGF-B and PlGF.

**Figure 6:**
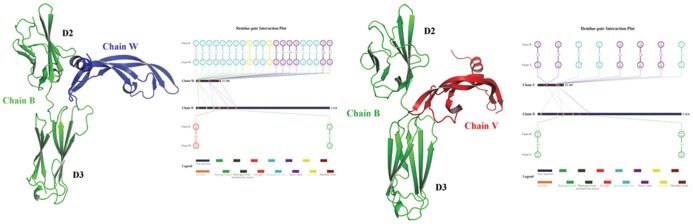
Schematic representation of diverse nonbonded interactions across the complex interface of VEGF-A chains W and V separately with conbercept chain B. This figure was prepared with the in-house program 2D-Gralab [51].

Further, the nonbonded interactions across the conbercept–VEGF-A binding interface were mapped onto 2D schematic plots using the in-house program 2D-Gralab [51]. As shown in Figure 6, the chemical forces that connect conbercept and VEGF-A are mainly nonspecific forces such as hydrophobic effects and steric contacts, rather than specific forces such as hydrogen bonding and salt bridges. The nonspecific force-driven interactions observed for conbercept with VEGF-A is typical for the recognition and association of immunoglobulin-like domains with their partners. These partners, mostly cell factors, are usually capable of interacting with a number of homologous receptors. More importantly, as compared with the complex crystal structure of VEGF-A with its cognate receptor VR1 D2 (PDB: 1 flt), the nonbonded interactions at the VEGF-A–conbercept D2 interface appear to be reduced, albeit the D2 region in VR1 and in conbercept are the same. This could be explained by the fact that fusing VR1 D2 and VR2 D3 together might undermine the compatibility between VEGF-A and VR1 D2, causing the reduction in affinity.

### Conclusions

For the first time, we herein described the extensive structural characterization of conbercept by instrumental analysis and molecular modeling. Orthogonal methods based on different physical and chemical separation properties such as mass spectrometry, high-performance liquid chromatography and capillary electrophoresis were applied to validate the structural model and identify possible anomalies. The structure was elucidated using the characterized protein containing the correct amino acid sequences and disulfide linkages. Based on the experimental results, the structure of the conbercept–VEGF-A complex was built using molecular modeling to explore the effects of structural variations on the protein binding and interactions. All the presented analytical methods and molecular modeling can be used during pharmaceutical development and for designing and characterizing promising therapeutic proteins. However, the MD-equilibrated structural model of conbercept–VEGF-A does not take into account the presence of oligosaccharides in conbercept. Oligosaccharide analysis of conbercept indicated that all seven N-glycosylation sites were occupied with complex oligosaccharides structures. The relatively large size of possible oligosaccharides could significantly change the overall protein structure and its proximity to the ligand binding site. Further studies will be required to build new conbercept–VEGF-A complex models, taking into consideration critical glycosylation modifications. This should help to understand how the presence of oligosaccharides in conbercept affect the binding affinity to VEGF-A. The model can also be applied for studying conbercept interactions with other ligands such as VEGF-B and PlGF.
